# ‘Pleomorphic adenoma of the tongue: A common entity at the uncommon location’

**DOI:** 10.1016/j.amsu.2018.12.004

**Published:** 2018-12-23

**Authors:** Sonia Chhabra, Namita Bhutani, Promil Jain, Meenu Gill, Nilay Nishith, Rajeev Sen

**Affiliations:** Deptt. of Pathology, PGIMS Rohtak, Haryana, India

**Keywords:** Adenoma, Pleomorphic, Tongue

## Abstract

The salivary gland tumors comprise of 3% of head and neck tumors. Pleomorphic adenoma, also known as mixed tumor, is the most common benign neoplasm of the major and minor salivary glands. The occurrence of pleomorphic adenoma of the tongue is very rare, and very few cases have been reported in the literature. A 41-year-old male presented with swelling on tongue. Cytological and histological examination revealed pleomorphic adenoma. Complete excision of the mass was done transorally under general anaesthesia. High index of suspicion and an adequate clearance of the tumor with a cuff of surrounding dispensable normal tissues is the key to successful treatment of such tumors. The authors consider the rarity of this case and present a rare case of pleomorphic adenoma of the tongue.

## Highlights

• The salivary gland lesions pose a challenge to even the most experienced clinician and pathologist.
• Pleomorphic adenomas of the tongue are very rare accounting for 0 to 2.5% of the cases.
• The high recurrence rate and frequent malignant conversion are its characteristics features.
• High index of suspicion and an adequate clearance of the tumor is the key to successful treatment of such tumors.

## 1 Introduction

Pleomorphic adenomas (PA) were first described by Minssen in Ahlbom's monograph in 1874 [1]. It is also called as mixed tumor and accounts for a total of 40%–70% of all major and minor salivary gland tumors [2]. PA's prime site of involvement is the parotid gland, and the minor salivary glands are the least involved. Among the minor salivary glands, PA of tongue is reported to be very rare accounting for 0–2.5% of the cases. In tongue the most common benign tumor is the pleomorphic adenoma whereas adenoid cystic carcinoma is the most common malignant tumor reported. Most of the studies indicate an overall ratio of approximately 1:6 for the benign/malignant lingual salivary gland tumors [3]. The high recurrence rate and frequent malignant conversion are its characteristics features. For most cases, the only clinical sign is an asymptomatic painless swelling with a slow growth rate. Microscopically, PA is characterised by variable, diverse structuring histological patterns consisting of fibrous, hyaline, myxoid, cartilaginous and osseous areas, which are differentiated by myoepithelial cells. The occurrence of pleomorphic adenoma of the tongue is very rare and till date very few cases have been reported in the literature [4,5]. The tumors of salivary gland constitute about 3% of all neoplasms [4]. The majority of salivary gland neoplasms are benign with pleomorphic adenomas (PA) being the most common. The incidence of salivary gland neoplasms in minor glands varies from 9 to 22%. Approximately 8% of pleomorphic adenomas occur in the minor salivary glands. The most frequent site of involvement is the palate (50%); followed by the lips and maxillary sinus [4,5]. Involvement of the tongue is extremely rare [1]. When they occur on the tongue, pleomorphic adenomas are most commonly seen in the posterior, followed by anterior, and rarely in the lateral lingual gland (Ebner's gland) of the tongue [7]. Malignant tumors such as adenocarcinoma, adenoid cystic carcinoma, and mucoepidermoid carcinoma involve the tongue more frequently than their benign counterparts [[3], [4], [5]]. We hereby report a rare case of pleomorphic adenoma of the tongue in a 41 years old male patient. The SCARE criteria were utilized for this case report [6].

## 2 Case report

**Fig. 1:**
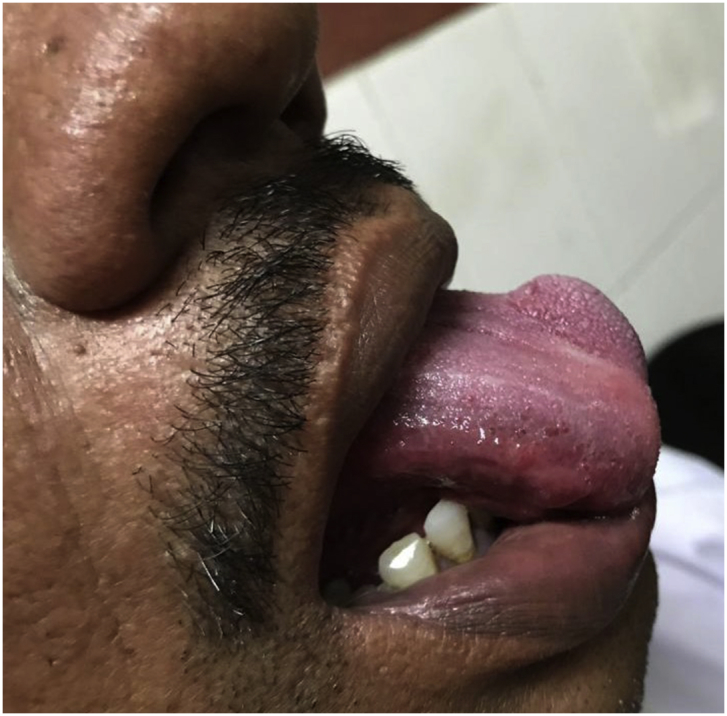
A solitary mass of size 2 × 1.4 cm was noted on left anterolateral aspect of tongue.

**Fig. 2:**
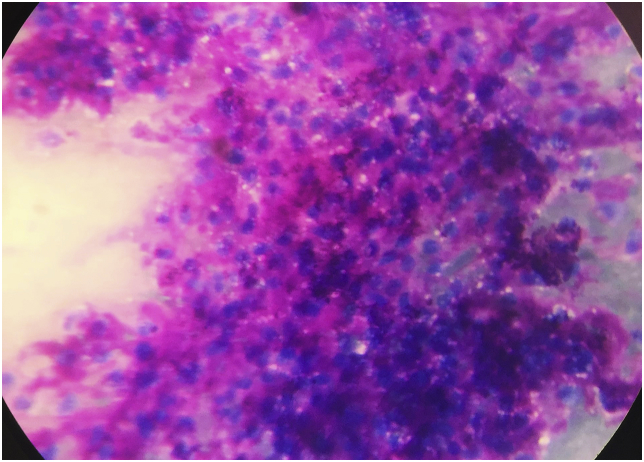
On FNAC benign epithelial cells embedded in dense eosinophilic chondromyxoid material (Leishman, 100X).

**Fig. 3:**
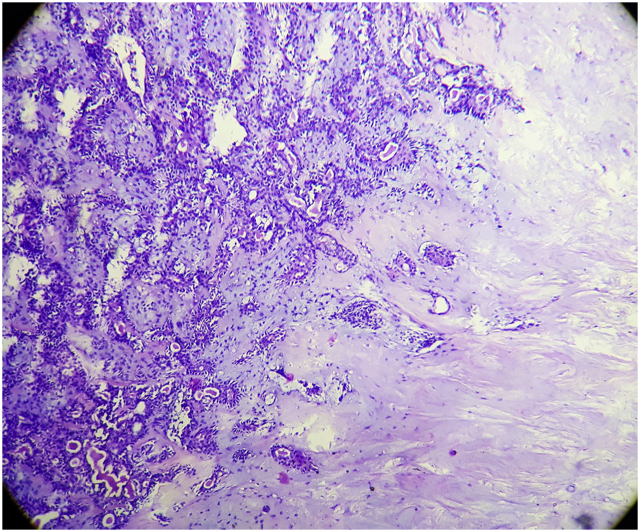
A tumor composed of islands and trabeculae of epithelial cells in chondromyxoid stroma (H & E, 100X).

A 41 years old male presented to the head and neck outpatient department with a complaint of a growth on the leftt side of the tongue that he noticed 25 days back. He also had dysphagia and difficulty in talking. He was a chronic smoker but there was no history of alcoholism. On local examination, an intraoral, submucosal, solitary mass of size 2 × 1.4 cm was noted on left anterolateral aspect of tongue (Fig. 1). It was initially small but has increased to the present size. There was no history of bleeding or any discharge from the growth or pain and sensory changes associated with it. Surface of the growth was smooth and erythematous with surrounding mucosa appearing normal. On palpation, the size and extent were confirmed. Growth was non-tender, firm in consistency, sessile, non-fluctuant, non-reducible, compressible and non-pulsatile. No evidence of blanching was observed on digital pressure. There was no history of bleeding on palpation. The ultrasonography of the lesion revealed a heteroechoic lesion of 2 × 1.4 cm with cystic change. Fine needle aspiration cytology (FNAC) of the mass was done which revealed round to oval benign epithelial cells embedded in dense eosinophilic chondromyxoid material (Fig. 2). Cytological features were suggestive of a benign salivary gland neoplasm favouring the diagnosis of pleomorphic adenoma. Subsequently, excision of the mass was performed under general anaesthesia. The histopathological examination of the specimen revealed a well encapsulated tumor composed of islands and trabeculae of epithelial cells in chondromyxoid stroma (Fig. 3). The postoperative period was uneventful and the patient was discharged on the 7th postoperative day and he is free of recurence till date.

## 3 Discussion

The incidence of pleomorphic adenoma peaks during the third decade of life and is more common in females than in males. Dysphagia is the most frequent initial symptom. The other symptoms can be pain, muffled phonation, and/or airway obstruction depending upon the size and location of the mass. They are slow growing; does not seem to change for many years and expresses itself simply as a nontender mass [7]. These are usually well demarcated or encapsulated but extension of tumor into the capsule is a common feature and sometimes lobules of tumor may appear to be completely separated from the main tumor mass. PA of intraoral accessory glands seldom is allowed to attain a size greater than 1–2 cm in diameter. Because this tumor causes the patient difficulties in mastication, talking and breathing, it is detected and treated earlier than other tumors of major glands. Imaging studies particularly CT and Magnetic resonance imaging (MRI) provides useful information on the nature and extent of the lesion, involvement of the lymph nodes, and the status of the surrounding tissues. MRI is preferred because of its ability to define the soft tissues with higher resolution [5].

Tumors of the salivary glands are of intricate origin consisting of epithelial and connective tissues. The origin of the pleomorphic adenoma is myoepithelial cells and intercalated duct cells [1]. The histopathological appearance of a pleomorphic adenoma is mainly composed of epithelial and myoepithelial elements, with a variety of patterns ending up embedded in mucopolysaccharidestroma. Fibrosis of the surrounding salivary parenchyma forms a capsule, usually false [1]. Pleomorphic adenoma of the minor salivary gland is known to have more cellular and fewer mesenchymal components. In cases of the elderly, malignant degeneration to carcinoma ex pleomorphic adenoma must be taken into consideration [5].

Treatment is primarily surgical under general anaesthesia, but depends on various parameters, such as age of the patient and size and site of the tumor. Although these tumors are well encapsulated, resection of the tumor with an adequate margin is essential to avoid recurrence [1]. The surgical approach depends on the size, site, and extent of the tumor. Surgical options include transoral, transcervical, midline transhyoid, transpharyngealand lateral pharngotomy approaches. When the tumor is malignant with extensive invasions into surrounding tissues, the latter two approaches are recommended [5]. Care must be taken to preserve the hypoglossal and lingual nerves. Use of carbon dioxide laser is becoming more widespread for resection. Recurrence is uncommon and may be attributed to partial excision or a multifocal origin of the tumor [8,9]. Some studies report a recurrence rate of 6% in patients with benign minor salivary gland tumors [1]. Approximately 20–45% of pleomorphic adenomas recur despite surgical excision and 2–9% will degenerate to malignant tumors. The risk of recurrence is increased if the capsule is torn. In elderly patients, malignant transformation of benign tumors of the minor salivary gland that is carcinoma-ex pleomorphic adenoma must always be taken into consideration. The follow-up of patients with salivary gland tumors should be long due to the possibility of late recurrences [10].

Differential diagnosis we considered due to the clinical presentation and localization of the lesion, included mucocele, reactive lesions such as giant cell fibroma or focal fibrous hyperplasia, lipoma, granular cell myoblastoma, neurofibroma, neurilemmoma, vascular leiomyoma, and benign salivary gland neoplasm. The diagnosis of these tumors is made on the basis of histopathological features. Incisional biopsy gives the pathologist a better chance of diagnosis than fine needle aspiration, without an increased morbidity. In the present case FNAC was done for the diagnosis. Recurrence is uncommon and may be due to partial excision or a multifocal origin of the tumor. Other rare tumors of the tongue are tuberculoma, amyloid tumor, mucoepidermoid carcinoma and angioleiomyoma [11,12].

## 4 Conclusion

The salivary glands may show a diverse range of lesions presenting a challenge to even the most experienced clinician and pathologist. Pleomorphic adenoma of tongue (minor salivary glands) is a tumor of rare occurrence. High index of suspicion and an adequate clearance of the tumor with a cuff of surrounding dispensable normal tissues is the key to successful treatment of such tumors.

## Provenance and peer review

Not commissioned, externally peer reviewed.

## Source of support

NIL.

## Ethical approval

Institutional review board approval was not required because all data were collected from clinical records.

## Sources of funding

Author declare there is no funding resources for this paper.

## Author contribution

DR. NAMITA BHUTANI WROTE THE PAPER.

DR. SONIA CHHABRA: REPORTED THE CASE.

DR. PROMIL JAIN: READ THE PAPER.

DR. RAJEEV SEN: ANALYSED THE PAPER.

## Conflicts of interest

The authors declare that there is no conflict of interest regarding the publication of this paper.

## Trial registry number

NOT APPLICABLE.

## Guarantor

DR. NAMITA BHUTANI and Dr Rajeev Sen.

## Appendix A Supplementary data

checklist checklist
